# Bare Neck and Arms

**Published:** 1879-12

**Authors:** 


**BARE NECK AND ARMS.**—There is, perhaps, not an eminent physician in any system of practice, who will not declare, with a distinguished medical practitioner, now deceased: "I believe, that during the twenty-six years I have followed my profession in this city, twenty thousand children have been carried to the cemeteries, a sacrifice to the absurd custom of exposing their arms naked."

### VARIOUS ITEMS.

"Exitur Numchais" is the post-office address on a letter; the child who can "make it out" may well be considered a youthful Daniel.

"The Pall Mall Gazette" ascribes the great power of John Bright over an audience to his large use of monosyllables in his speeches. In a grand passage of his speech on the Burials bill describing a Quaker funeral, out of 190 words 149 were of a single syllable.

